# Optical Coherence Tomography Image Enhancement and Layer Detection Using Cycle-GAN

**DOI:** 10.3390/diagnostics15030277

**Published:** 2025-01-24

**Authors:** Ye Eun Kim, Eun Ji Lee, Jung Suk Yoon, Jiyoon Kwak, Hyunjoong Kim

**Affiliations:** 1 Department of Statistics and Data Science, Yonsei University, Seoul 03722, Republic of Korea; kyeun0628@yonsei.ac.kr; 2 Department of Ophthalmology, Seoul National University College of Medicine, Seoul National University Bundang Hospital, Seongnam 13620, Republic of Korea; opticdisc@gmail.com (E.J.L.); jungseok91@gmail.com (J.S.Y.); 1jiyoonk1@gmail.com (J.K.)

**Keywords:** optical coherence tomography (OCT), retinal nerve fiber layer (RNFL), generative adversarial networks (GANs), pix2pix, cycle-GAN

## Abstract

**Background/Objectives:** Variations in image clarity across different OCT devices, along with the inconsistent delineation of RNFL boundaries, pose a challenge to achieving consistent diagnoses for glaucoma. Recently, deep learning methods such as GANs for image transformation have been gaining attention. This paper introduces deep learning methods to transform low-clarity images from one OCT device into high-clarity images from another, concurrently estimating the retinal nerve fiber layer (RNFL) segmentation lines in the enhanced images. **Methods:** We applied two deep learning methods, pix2pix and cycle-GAN, and provided a comparison of their performance by evaluating the similarity between the generated and actual images, as well as comparing the generated RNFL boundary delineation with the actual boundaries. **Results:** The image conversion performance was compared based on two criteria: Fréchet Inception Distance (FID) and curve dissimilarity. In the comparison of FID values, the cycle-GAN method showed significantly lower values than the pix2pix method (*p*-value < 0.001). In terms of curve similarity, the cycle-GAN method also demonstrated higher similarity to the actual curves compared to both manually annotated curves and the pix2pix method (*p*-value < 0.001). **Conclusions:** We demonstrated that the cycle-GAN method produces more consistent and precise outcomes in the converted images compared to the pix2pix method. The resulting segmented lines showed a high degree of similarity to those manually annotated by clinical experts in high-clarity images, surpassing the boundary accuracy observed in the original low-clarity scans.

## 1. Introduction

Quantitative measurement of retinal nerve fiber layer (RNFL) thickness around the optic disk, facilitated by optical coherence tomography (OCT), is essential for diagnosing and monitoring glaucoma. However, a significant limitation arises due to discrepancies in diagnostic outcomes across different OCT devices. These variations stem from differences in image clarity and the proprietary algorithms used to measure RNFL thickness. This lack of compatibility between devices presents challenges in clinical communication across institutions with different equipment, impeding the transfer system and substantially impacting disease diagnosis and patient follow-up. Consequently, a solution that enhances device compatibility is urgently needed.

### 1.1. Related Work

In the field of computer vision, the issue of image data transformation is a very important topic, and many deep learning methods have been developed. Since the introduction of generative adversarial networks (GANs) [1], a widely recognized deep learning method for image conversion, numerous image processing techniques utilizing GANs have emerged. These methods include tasks such as enhancing image resolution [2], manipulating images by adjusting latent variables [3,4,5,6], and blending the styles of two distinct images [7,8,9]. Among these, image-to-image translation involves transforming an input image into another image with a specified target style. For example, this includes converting grayscale photos to color images or transforming horse images into zebra images.

In the field of ophthalmology, some studies have been presented on transforming OCT images into new images using GAN techniques. For example, a method for drusen segmentation in en face OCT images was proposed by combining the pix2pix method, a type of GAN, with an embossing technique [10]. A method using cycle-GAN for OCT image-to-image translation was presented to reduce domain differences [11]. A 3D cycle-GAN approach was proposed to translate retinal OCT images into confocal microscopy images [12]. An optimized GAN framework for generating realistic OCT B-scan images of diabetic macular edema (DME) was introduced [13]. An unsupervised unpaired learning approach was presented to reduce image variability across different OCT devices [14].

### 1.2. Contribution

One of the objectives of this study is to establish a method for transforming OCT images captured with Cirrus spectral-domain OCT (SD-OCT; Zeiss, Oberkochen, Germany) into clearer images, similar to those obtained with Spectralis SD-OCT (Heidelberg Engineering, Heidelberg, Germany). Another objective is to automatically generate RNFL boundary lines in the transformed images that resemble those drawn by clinical experts. To achieve the second objective, the model must also learn to create RNFL boundary lines during the image transformation process. We employed a deep learning model called GANs for image-to-image translation. Specifically, we present an application of pix2pix and cycle-GAN methods, which are types of GANs, enabling the generation of high-clarity images with RNFL segmentation lines.

No method has yet been attempted to directly estimate lines that delineate the RNFL boundaries. In this study, we aim to generate RNFL boundary lines simultaneously with image conversion. This approach sets our work apart from previous studies.

## 2. Materials and Methods

**Figure 1:**
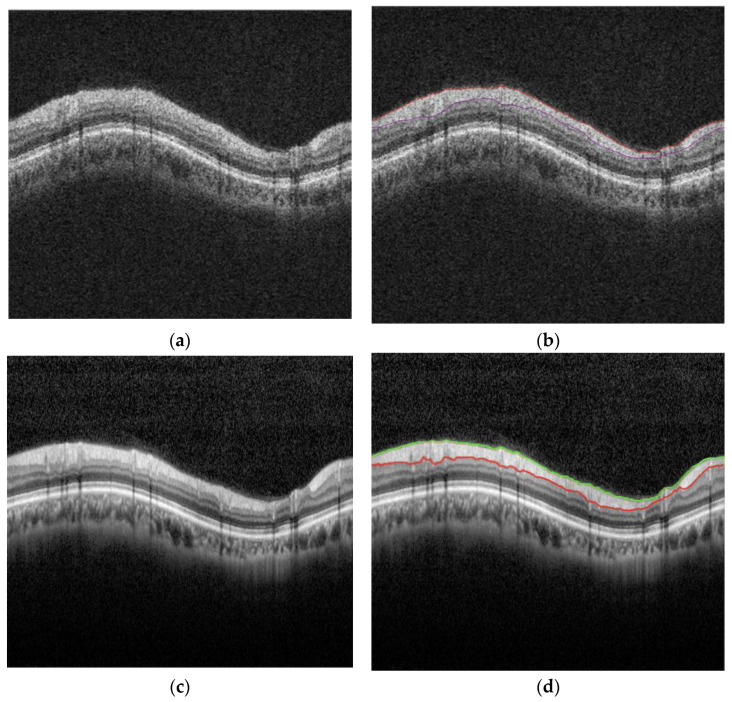
Four types of image datasets. (**a**) A-raw; (**b**) A-curve; (**c**) B-raw; (**d**) B-curve. The colored lines are the manual segmentation lines by clinical experts.

The OCT image dataset used in this study was obtained from 244 eyes, all of which belong to either glaucoma patients or glaucoma suspects. For each eye, images were acquired from two of the most widely used OCT devices in ophthalmology: Zeiss’s Cirrus SD-OCT (224 images, referred to as A-raw) and Heidelberg’s Spectralis SD-OCT (244 images, referred to as B-raw). Additionally, manual segmentation of the RNFL was performed by clinical experts on both the A-raw and B-raw images, referred to as A-curve (167 images) and B-curve (244 images), respectively. The A-raw, A-curve, B-raw and B-curve image data are four-way paired because they are captured from the same eye of the same patient. Note that there are 77 missing images in the A-curve dataset, as cases with segmentation errors due to equipment image quality issues were excluded. Figure 1 shows examples of these four types of images.

This study aims to develop an effective deep learning model capable of directly transforming A-raw images into B-curve images. Therefore, only A-raw and B-curve images need to be used in the training phase. This means that B-raw and A-curve images will not be used during the model development process. However, A-curve images will later be used as a baseline in the evaluation phase for assessing the model’s image conversion capability.

### 2.1. GAN Model

Generative adversarial networks (GANs) is a deep learning method that have made the most significant contributions to the field of image translation. Since the methods used in this study are also derived from GANs model, it is necessary to briefly summarize the GANs model first.

A typical GANs model uses a pair of generator $G_{X\to Y}$ and discriminator $D_{Y}$, which are implemented as deep neural networks. The generator $G_{X\to Y}$ creates an image $\hat{Y}$, while the discriminator $D_{Y}$ tries to distinguish between the generated image $\hat{Y}$ and the true image Y. The generator $G_{X\to Y}$ strives to win the discriminator $D_{Y}$ by producing more realistic images, while the discriminator $D_{Y}$ tries to win the generator $G_{X\to Y}\;$ by successfully identifying the generated images from true image. This adversarial relationship stimulates both models to improve simultaneously, ultimately enabling the generation of more refined and realistic images.

The loss function for training GANs framework is defined as follows:

$$L_{GAN}\left(G,D\right)=L_{GAN}\left(D_{Y}\right)+L_{GAN}\left(G_{X\to Y}\right),$$

where $L_{GAN}\left(D_{Y}\right)$ is the loss function of $D_{Y}$ and $L_{GAN}\left(G_{X\to Y}\right)$ is the loss function of $G_{X\to Y}$. $L_{GAN}\left(D_{Y}\right)$ evaluates the extent to which $D_{Y}$ is unable to distinguish between real and generated images. As the generated images become increasingly similar to real images, $L_{GAN}\left(G_{X\to Y}\right)$ decreases, reflecting its effectiveness in deceiving the discriminator $D_{Y}$.

Finally, the GANs model finds an optimal generator $G^{*}$ by solving the min-max problem as described in the following equation:

$$G^{*}=\arg\min_{G}\max_{D}\left[L_{GAN}\left(G,D\right)\right].$$

Since the A-raw and B-curve images are taken from the same patient’s eye, they can be considered paired. Therefore, a deep learning method that can handle transformations between paired images is needed. Among the GAN-based deep learning methods, pix2pix and cycle-GAN are suitable for transforming paired OCT images. We present explanations of the two methods used in this paper in the following sections.

### 2.2. Pix2pix Method

The pix2pix method [15] is a conditional GANs that uses the input image as a conditioning variable. It operates by pairing an input image X with a corresponding target image Y at the pixel level, enabling paired image-to-image conversion. This model can perform various paired image conversion tasks, such as converting sketches into realistic images or transforming satellite photos into map images.

In the pix2pix method, another loss function called consistency loss needs to be included, which is defined below.

$$L_{con}\left(G_{X\to Y}\right)=E\left(\left\|Y-\hat{Y}\right\|\right).$$

Its exact spatial arrangement of the input image is preserved through the consistency loss because it regularizes the resulting translation $G_{X\to Y}$ to produce images that closely resemble the target image at the pixel level. Without this consistency loss, the generator $G_{X\to Y}$ may map any X to a single well-transformed image, losing the specific correspondence between input and target images.

Finally, the generator $G_{X\to Y}$ is trained to minimize the loss function, while the discriminator $D_{Y}$ is trained to maximize the loss by distinguishing between real and generated images. Consequently, the adversarial learning process aims to find an optimal generator $G^{*}$ by solving the min-max problem as described in the following equation:

$$G^{*}=\arg\min_{G}\max_{D}\left[L_{GAN}\left(G,D\right)+\eta L_{con}\left(G_{X\to Y}\right)\right].$$

However, the pix2pix method has a limitation in that it only performs well when perfectly paired image data at the pixel level is available.

### 2.3. Cycle-GAN Method

The cycle-GAN method [16] is an unpaired image-to-image translation framework designed for scenarios where two image sets, X and Y, are not directly paired. This approach is particularly useful when the transformation between X and Y  is complex and cannot be matched through simple operations. For instance, when transforming a horse into a zebra, it is often impossible to obtain directly paired images. The reason the word ’cycle’ is used in cycle-GAN is that the model is trained to generate $\hat{Y}$ from an X image, and then to cycle back by transforming $\hat{Y}$ into an image similar to X.

In cycle-GAN, two generators, $G_{X\to Y}$ and $G_{Y\to X}$, and two discriminators, $D_{Y}\;$ and $D_{X}$, are employed. The generator $G_{X\to Y}$ learns to transform images from X to resemble those in Y, while $G_{Y\to X}\;$performs the inverse transformation. Discriminators $D_{Y}\;$and $D_{X}$ are tasked with evaluating the similarity between the generated images and the original images from their respective domains.

A key innovation in cycle-GAN is the introduction of cycle-consistency loss:

$$L_{cyc}\left(G_{X\to Y},G_{Y\to X}\right)=E\left(\left\|X-G_{Y\to X}(\hat{Y})\right\|\right)+E\left(\left\|Y-G_{X\to Y}(\hat{X})\right\|\right).$$

This loss function enables learning in unpaired image settings where a direct correspondence between input and output images is unavailable. By transforming an input image from one domain into another, $\hat{Y}=G_{X\to Y}(X)$, and then reconverting it back into the original domain, $G_{Y\to X}(\hat{Y})$, the cycle-consistency loss ensures that the resulting image is reversible and retains the essential characteristics of the original input. This mechanism guarantees that the generated image closely resembles the initial input image after the cycle transformation.

Without the cycle-consistency loss, the generator might produce images that are unrelated to the input, as the discriminator evaluates the generated images solely based on their plausibility rather than their correspondence with the input. For example, during the task of transforming a horse image into a zebra image, the generator could convert a sitting horse into a standing zebra, leading to inconsistencies. The cycle-consistency constraint mitigates this issue by enforcing structural consistency between the input and output.

Ultimately, the optimal generators, ${G_{X}}^{*}$ and ${G_{Y}}^{*}$ are obtained by solving the min-max problem through adversarial training, as described below.

$${G_{X}}^{*},{G_{Y}}^{*}=\arg\min_{G}\max_{D}\left[L_{GAN}\left(G_{X\to Y},D_{Y}\right)+L_{GAN}\left(G_{Y\to X},D_{X}\right)+\lambda L_{cyc}\left(G_{X\to Y},G_{Y\to X}\right)\right].$$

One point to note is that the cycle-GAN method can also be used with paired data. Since paired data are even more similar than unpaired data, they may be easier to fit a cycle-GAN model, so it is expected to work naturally well with paired image data at the pixel level.

### 2.4. Semi-Paired Image Data

The X and Y used in the descriptions of Section 2.1 and Section 2.2 refer to A-raw and B-curve image data, respectively. Although the OCT image sets are obtained from the same eye of the same patient, there may be slight differences in object positioning and shape between the A-raw and B-curve images due to the effects of different devices. While the overall curvature and RNFL thickness patterns may appear similar, they do not align perfectly when the two images are overlaid. We refer to these cases as semi-paired images, where the images represent the same object but are not perfectly aligned at the pixel level.

When using pix2pix, the results may not be optimal with semi-paired images due to the lack of precise pixel-level correspondence. In contrast, applying the cycle-GAN transformation method to semi-paired images can yield better performance, as semi-paired image data are easier for cycle-GAN model training than entirely dissimilar image data. To validate this, this paper seeks to transform images using both methods and compare the outcomes across some performance metrics.

## 3. Results

### 3.1. Experiment Settings

We divided the dataset of 244 semi-paired images into 195 training and 49 test datasets. To ensure a fair comparison between the pix2pix and cycle-GAN methods, both models were trained using the same training data and evaluated on the same test data.

For training both methods, a learning rate of 0.0002 and the Adam optimizer with β_1_ = 0.5 were used. Drop-out with a rate of 0.5 was applied to prevent overfitting, and batch normalization was employed to stabilize the training process. Each method was trained for 100 epochs, with training images randomly cropped and horizontally flipped to provid threefold data augmentation. For a reliable comparison of the models’ performances, training was repeated 10 times. To assess the image conversion performance of the developed pix2pix and cycle-GAN models, we used 49 paired test data.

The evaluation process using test data is as follows. First, the A-raw image from the test data is put into the already trained model to obtain a generated B-curve image, denoted as $\hat{B}$-curve. Then, the $\hat{B}$-curve image is compared with the actual B-curve image to evaluate how closely the prediction resembled the actual one.

### 3.2. Image Conversion Result

**Figure 2:**
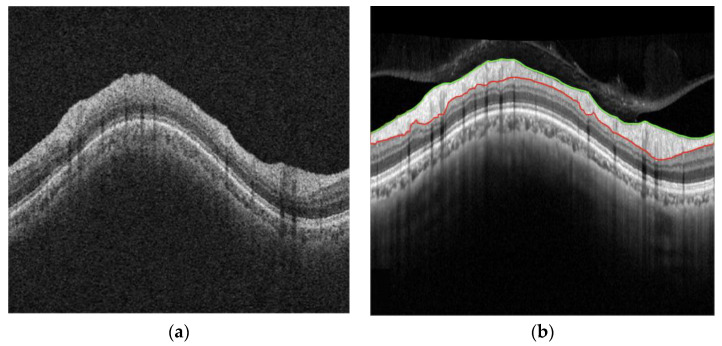
Images of A-raw and B-curve. (**a**) A-raw; (**b**) B-curve. The colored lines are the manual segmentation lines by clinical experts.

**Figure 3:**
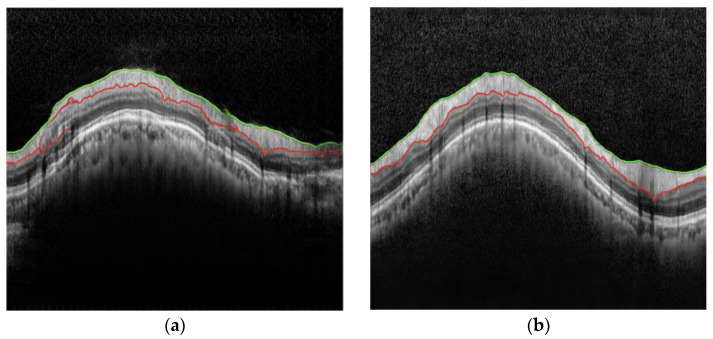
Predicted $\hat{B}$-curve images by transforming the A-raw image. (**a**) Pix2pix; (**b**) cycle-GAN. The colored lines are the segmentation lines generated by each method.

Figure 2 shows one of the A-raw and B-curve images used in the test stage, while Figure 3 presents the $\hat{B}$-curves by transforming the A-raw image using the pix2pix and cycle-GAN methods, respectively. When compared to the actual B-curve image data, the pix2pix method struggled to produce accurate images. Due to the semi-paired images not aligning perfectly at the pixel level, pix2pix generated outputs with several overlapping ghost images. In contrast, cycle-GAN was able to generate $\hat{B}$-curve images that more closely resembled the B-curve.

### 3.3. Quantitative Evaluation

Since it is important to measure the reproduction performance of segmentation boundaries in OCT images, quantitative evaluation methods should take this aspect into account. From this perspective, we intend to use Fréchet Inception Distance (FID) and curve dissimilarity. FID, unlike simpler metrics (e.g., pixel-wise differences), captures perceptual and structural similarities between two images, while curve dissimilarity is particularly suitable for evaluating the accuracy of the segmentation line.

#### 3.3.1. FID (Fréchet Inception Distance)

The Fréchet Inception Distance (FID) serves as a metric that quantifies the similarity in esthetic or style between ground truth images and the generated images [17]. A lower FID value signifies a closer alignment between the converted images and the true images. Table 1 illustrates the FID values calculated between the predicted $\hat{B}$-curve and the actual B-curve, providing a quantitative measure of their similarity.

Among the evaluated methods, cycle-GAN achieved a lower FID value than pix2pix, with the *p*-value indicating a significant difference between them.

#### 3.3.2. Curve Dissimilarity

Assuming the curves in the actual B-curve image represent the ground truth, we expect the lines of the $\hat{B}$-curve image to closely resemble those in the actual B-curve image. To assess the similarity in RNFL thickness between the $\hat{B}$-curve image and the actual B-curve image of the same eye, we define the curve dissimilarity of an image as follows.

Let the function c(·) represent the relative thickness of RNFL, calculated based on the horizontal pixel positions in the OCT image. This function takes an image with corresponding segmentation lines and computes the width between the upper and lower segmentation lines at each horizontal pixel, returning a vector of length equal to the image’s width. That is, $c\left(X\right)=s_{1}(X)-s_{2}(X)$, where $s_{1}$ and $s_{2}$ are the upper and lower segmentation lines, respectively.

**Figure 4:**
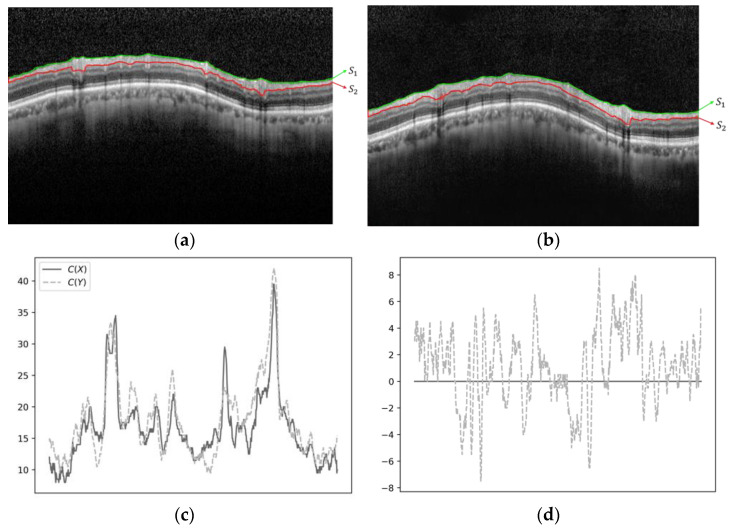
Example of c(X) and c(Y) and their differences. (**a**) Upper and lower layers of X; (**b**) upper and lower layers of Y; (**c**)$\;c\left(X\right)$ and $c\left(Y\right)$; (**d**) $c\left(X\right)-c\left(Y\right)$.

The curve dissimilarity h(X,Y) is then defined as the square root of the mean of the squared differences between c(X) and c(Y), expressed mathematically as

$$h\left(X,Y\right)=\sqrt{\frac{1}{p}{\left\|c\left(X\right)-c(Y)\right\|}^{2}},$$

where ${\left\|\cdot \right\|}^{2}\;$denotes the $L_{2}$ norm, and p denotes the total number of horizontal pixels in the images X and Y. Figure 4 provides an example illustrating the functions c(X) and c(Y) and their differences.

Note that A-curves refer to images manually delineated RNFL by clinical experts on A-raw images. In this section, we additionally utilize the A-curve image data to include a comparison between the segmentation line on the A-curve and the B-curve. Since some of the test data have missing A-curve images, we will perform the comparative evaluation using 36 semi-paired images with no missing data instead of the full 49 test images.

Table 2 presents the curve dissimilarity, h(A-curve, B-curve) and $h(\hat{B}$-curve, B-curve) on 36 test images. Notably, the cycle-GAN method achieved lower curve dissimilarity values than the A-curve, indicating that the cycle-GAN converted lines were more accurate than human annotations on low-quality images.

According to Table 2, the method with the lower curve dissimilarity values on test images was cycle-GAN. This indicates that the cycle-GAN produces results more like the lines in B-curve images than pix2pix.

To statistically verify the paired differences between these methods, confidence intervals and *p*-values were calculated using the ANOVA (Randomized Complete Block Design) method using images as blocks. An examination of the C.I and *p*-values shows that cycle-GAN achieved superior results compared to pix2pix.

## 4. Discussion

This study aimed to improve compatibility between images from two different OCT devices by training a GAN model with the goals of not only transforming one OCT image into another but also generating lines that delineate the RNFL boundaries that are essential for glaucoma diagnosis. Among GAN models, we considered the pix2pix method, which can be used with paired OCT images, and the cycle-GAN method, which can be used regardless of whether the images are paired.

OCT image data were taken from 244 eyes. For each eye, images were acquired using two OCT devices: Zeiss’s Cirrus SD-OCT (referred to as A-raw) and Heidelberg’s Spectralis SD-OCT (referred to as B-raw). Additionally, manual segmentation of the RNFL was performed by clinical experts on both the A-raw and B-raw images, referred to as A-curve and B-curve, respectively. Then, OCT image datasets were randomly divided into 195 training datasets and 49 test datasets.

Two GAN models, pix2pix and cycle-GAN, are trained using the same training data. They are designed to directly transform A-raw images into B-curve images. Therefore, only A-raw and B-curve images need to be used in the training phase. A training strategy that involves transforming A-raw into A-curve or B-raw, and subsequently into B-curve, could be considered. However, this approach not only requires longer training time but also carries the risk of error accumulation at each stage. Therefore, we determined that directly transforming A-raw into B-curve by skipping intermediate steps would be more effective.

Although the training images data were paired eye-wise, the objects within each pair did not perfectly align at the pixel level due to differences in object positioning and shapes within the images. We refer to this type of data as semi-paired data. Semi-paired data led to issues when applying the paired image conversion method, pix2pix. In contrast, the unpaired translation approach, cycle-GAN, achieved successful image conversion.

Using semi-paired test data, the image conversion performance was compared based on two criteria: Fréchet Inception Distance (FID) and curve dissimilarity. While all 49 test datasets were used for comparing FID, only 36 test datasets were used for comparing curve dissimilarity due to missingness in the A-curve datasets.

According to the FID and curve dissimilarity values, the cycle-GAN method showed lower values than the pix2pix method. This indicates that the cycle-GAN has better conversion performance. Furthermore, in RNFL detection, cycle-GAN showed superior accuracy over A-curve images, where curves were manually annotated by clinical experts.

Some studies [10,11,12,13,14] have aimed to enhance the quality of OCT images using cycle-GAN. However, no method has yet been attempted to directly estimate lines that delineate the RNFL boundaries. This study can be distinguished from others in that it generates RNFL boundary lines after conversion from A-raw to $\hat{B}$-curve image.

A limitation of this study is that the analysis was conducted using only 244 image sets, as we retrospectively investigated cases where images from both devices were available; however, collecting more image data from many devices could lead to more stable training results. In this study, patient demographic characteristics or pathological variability are not considered, as it is difficult to account for such factors when applying deep learning methods using OCT image data.

## Figures and Tables

**Table 1:** FID and their significances.

Methods	FID (m±sd)	C.I.	*p*-Value
pix2pix	90.08±0.72	(89.35, 90.81)	<0.001
cycle-GAN	86.78±0.95	(86.05, 87.51)	

**Table 2:** Curve dissimilarity and statistical significances by ANOVA.

Methods	Curve Dissimilarity	C.I.	*p*-Value with Cycle-GAN
A-curve	12.84	(12.39, 13.29)	<0.001
pix2pix	32.13±15.55	(30.17, 34.09)	<0.001
cycle-GAN	9.06±4.26	(8.61, 9.51)	

## Data Availability

The datasets generated during and/or analyzed during the current study are available from the corresponding author upon reasonable request.
